# Dexamethasone Predisposes Human Erythroblasts Toward Impaired Lipid Metabolism and Renders Their *ex vivo* Expansion Highly Dependent on Plasma Lipoproteins

**DOI:** 10.3389/fphys.2019.00281

**Published:** 2019-04-04

**Authors:** Maria Zingariello, Claudio Bardelli, Laura Sancillo, Fiorella Ciaffoni, Maria Luisa Genova, Gabriella Girelli, Anna Rita Migliaccio

**Affiliations:** ^1^Unit of Microscopic and Ultrastructural Anatomy, Department of Medicine, University Campus Bio-Medico, Rome, Italy; ^2^Department of Biomedical and NeuroMotor Sciences, Alma Mater Studiorum University, Bologna, Italy; ^3^Core Facilities – Istituto Superiore di Sanità, Rome, Italy; ^4^Centro Trasfusionale, Sapienza University of Rome, Rome, Italy

**Keywords:** erythropoiesis, lipid metabolism, dexamethasone, *ex vivo* expansion, transfusion products

## Abstract

Cultures of stem cells from discarded sources supplemented with dexamethasone, a synthetic glucocorticoid receptor agonist, generate cultured red blood cells (cRBCs) in numbers sufficient for transfusion. According to the literature, however, erythroblasts generated with dexamethasone exhibit low enucleation rates giving rise to cRBCs that survive poorly *in vivo*. The knowledge that the glucocorticoid receptor regulates lipid metabolism and that lipid composition dictates the fragility of the plasma membrane suggests that insufficient lipid bioavailability restrains generation of cRBCs. To test this hypothesis, we first compared the expression profiling of erythroblasts generated with or without dexamethasone. This analysis revealed differences in expression of 55 genes, 6 of which encoding proteins involved in lipid metabolism. These were represented by genes encoding the mitochondrial proteins 3-Hydroxymethyl-3-Methylglutaryl-CoA lyase, upregulated, and 3-Oxoacid CoA-Transferase1 and glycerol-3-phosphate acyltransferase1, both downregulated, and the proteins ATP-binding cassette transporter1 and Hydroxysteroid-17-Beta-Dehydrogenase7, upregulated, and cAMP-dependent protein kinase catalytic subunit beta, downregulated. This profiling predicts that dexamethasone, possibly by interfering with mitochondrial functions, impairs the intrinsic lipid metabolism making the synthesis of the plasma membrane of erythroid cells depend on lipid-uptake from external sources. Optical and electron microscopy analyses confirmed that the mitochondria of erythroblasts generated with dexamethasone are abnormal and that their plasma membranes present pebbles associated with membrane ruptures releasing exosomes and micro-vesicles. These results indicate that the lipid supplements of media currently available are not adequate for cRBCs. To identify better lipid supplements, we determined the number of erythroblasts generated in synthetic media supplemented with either currently used liposomes or with lipoproteins purified from human plasma [the total lipoprotein fraction (TL) or its high (HDL), low (LDL) and very low (VLDL) density lipoprotein components]. Both LDL and VLDL generated numbers of erythroid cells 3-2-fold greater than that observed in controls. These greater numbers were associated with 2–3-fold greater amplification of erythroid cells due both to increased proliferation and to resistance to stress-induced death. In conclusion, dexamethasone impairs lipid metabolism making *ex vivo* expansion of erythroid cells highly dependent on lipid absorbed from external sources and the use of LDL and VLDL as lipid supplements improves the generation of cRBCs.

## Introduction

The progress recently made in the development of culture conditions that allow generating great numbers of cultured red blood cells (cRBCs) from discarded stem cell sources is prompting numerous studies aimed to validate these cells as alternative transfusion products (Anstee et al., 2012; Bouhassira, 2012; Migliaccio et al., 2012). Several investigators are currently addressing the numerous challenges encountered in bringing cRBCs into the clinic. The proof-of-principle in animal models was provided in 2008 by the Nakamura laboratory which indicated that transfusion of *ex vivo* generated erythroblasts protects mice from lethal hemolytic anemia (Hiroyama et al., 2008). Giarratana et al. (2011) provided the first proof-of-concept in man by demonstrating that autologous cRBCs generated from mobilized CD34^pos^ cells survive for approximately 30 days *in vivo*. The Douay study also defined good manufacturing practice (GMP) conditions and minimal safety criteria of cRBCs for transfusion (Giarratana et al., 2011; Clinicaltrials.gov, 2012). Additional steps forward were represented by the demonstration that these cells express normal levels of blood group antigens and remain viable after cryopreservation for at least 8 years. Furthermore, these cells may also be generated from discarded buffy coats from blood donations with rare blood phenotypes collected under GMP conditions using media composed by clinical grade reagents of human origin and may be exchanged among laboratories across countries without loss of viability (reviewed in Zeuner et al., 2012).

The development of culture conditions allowing generating great numbers of cRBCs was pioneered by Dr. Fibach who was the first to divide the cultures into two phases (Fibach et al., 1989): the first phase (proliferation) induces hematopoietic stem/progenitor cells to generate morphologically recognizable erythroblasts; the second phase maturation sustains terminal maturation and enucleation generating cRBCs. The two phases are promoted by two different sets of stimuli. In the first phase, the signaling supporting more efficiently proliferation mimics stress erythropoiesis. These conditions were devised by exploiting the knowledge that, *in vivo*, recovery from acute anemia requires activation of the stress pathway controlled by the glucocorticoid receptor that confers to erythroid progenitors the ability to self-replicate (von Lindern et al., 1999; Dolznig et al., 2006; Zhang et al., 2013). Cultures that mimic the stress pathway are therefore stimulated with hematopoietic cytokines (stem cell factor, SCF; interleukin-3, IL-3; and erythropoietin, EPO) in combination with synthetic agonists of the glucocorticoid receptor (either hydrocortisone or dexamethasone, Dex) (Migliaccio et al., 2002; Giarratana et al., 2005; Leberbauer et al., 2005). Maturation is scanty in proliferation cultures. To promote maturation, cells generated after 12–15 days of proliferation cultures are transferred to the second phase culture stimulated with EPO where the cells mature in 7–10 days.

For reasons only partially known, erythroblasts generated with Dex mature in great numbers *in vivo* when transfused into immune-deficient hosts but enucleate with low efficiency when transferred in maturation cultures (Neildez-Nguyen et al., 2002). This challenge was first addressed by the discovery that maturation of erythroblasts into cRBCs is greatly improved by the presence of a feeder layer (Neildez-Nguyen et al., 2002). Since the feeder cells are often of animal original, these conditions are poorly suited for production of clinical grade products. Following studies discovered that feeder cells may be replaced by mifepristone, an antagonist of the glucocorticoid receptor, and plasmanate, a clinical grade derivative of human plasma (Miharada et al., 2006). In addition, enucleation rates may be further improved, reaching approximately 90%, by supplementing the maturation cultures with unfractionated human plasma and thyroid hormone (van den Akker et al., 2010; Masiello et al., 2013). These observations suggest that enucleation *in vitro* is promoted by inhibition of the glucocorticoid receptor signaling, activation of signaling from the thyroid hormone receptor and by components still to be identified present in human plasma. While genetic studies in mice have clarified that the thyroid hormone receptor exerts a pivotal role in the regulation of terminal erythroid maturation (Tanabe et al., 2007), the roles exerted in this process by the glucocorticoid receptor and by human plasma are still poorly understood.

The observation that survival of cRBCs *in vivo* (30 days) is inferior to that expected for young cells (120 days) (Giarratana et al., 2011) suggests that even under the best enucleation conditions developed up to now, cRBCs are fragile. Since the fragility of RBCs is greatly dependent on the physical-chemical properties of their plasma membrane, including fluidity, which are in turn regulated by its lipid composition (Brewer, 1980; An and Mohandas, 2008; Pollet et al., 2018), we hypothesized that the presence of Dex impairs the lipid metabolism of erythroid cells making the biosynthesis of their membranes extremely dependent on lipid uptake from lipoproteins present in human plasma and supplemented in the culture medium.

The plasma membrane of erythroid cells, as that of all the other cell types, consists of a lipid-bilayer attached to the cytoskeleton by integral membrane proteins (Mohandas and Gallagher, 2008). Approximately half of the mass of a RBC is represented by lipids (primarily phospholipids and non-esterified cholesterol) the balanced composition of which has been demonstrated to confer resistance to sheer stress in the circulation (Brewer, 1980; An and Mohandas, 2008; Pollet et al., 2018). *In vivo*, erythroblasts obtain their lipids either by biosynthesis or by absorption from their natural carriers, the circulating lipoproteins, present in plasma (van den Broek et al., 2017). In culture, erythroblasts may synthesize their lipids from fatty acids carried by albumin and absorb lipids by uptake either from lipoproteins present in fetal bovine serum (FBS) or from synthetic liposomes composed by bovine or human albumin, egg cholesterol and soybean lecithin (Migliaccio and Migliaccio, 1987). Therefore, the lipid composition, and integrity, of the plasma membrane of cultured erythroblasts depends both on the efficiency of the intrinsic cell biosynthetic pathways of the cell and on the presence in the media of lipid carriers suited for optimal absorption (Huang et al., 2018). To a surprise, in spite of the well-established effects exerted by glucocorticoids on lipid metabolism in other systems (Patel et al., 2014), studies to clarify lipid metabolism in erythroid cells and how it is affected by glucocorticoid receptor agonists are scanty.

The “research question” of this study was to clarify the effects exerted by lipid supplements during all the stages of erythroid maturation from progenitor cells down to mature cells using multiple end-points. The aim of this question was to formulate a culture media containing lipid supplements designed for erythroid cells. We hypothesized that the use of this media would allow generating greater numbers of human erythroblasts in proliferation culture. These erythroblasts would also have greater potential for terminal maturation. To address this research question, we first compared the expression profiling of erythroblasts generated with and without Dex. Next we evaluated the morphology of the plasma membranes and of the mitochondria of proerythroblasts generated with Dex and lastly we performed a comprehensive analysis of the effects exerted by various lipid supplements on the number and quality of the erythroid cells generated in culture. The results obtained indicate that Dex impairs the metabolism and membrane homeostasis of the erythroblasts making their expansion and maturation exquisitely dependent from lipids uptake from exogenous sources.

## Materials and Methods

### Human Specimens and Cell Preparation

Plasma and buffy-coats from whole blood donations were discarded material provided from Centro Trasfusionale, University of La Sapienza to be used in experiments performed at Istituto Superiore Sanità. The consent form of the Centro Trasfusionale includes consent to the use of the donation for research. Since specimens were provided as de-identified material, the study was considered non-human subject research by the ethical committee of Istituto Superiore Sanità. Buffy-coats were subjected to mononuclear cell (MNC) separation by Ficoll-Paque (Sigma, St. Louis, MO, United States) centrifugation, cryopreserved in Iscove’s modified Dulbecco’s medium (IMDM, Gibco-Invitrogen, Carlsbad, CA, United States), FBS (50% vol/vol, Sigma) and dimethylsulphoxide (10% vol/vol, Sigma) and stored in liquid nitrogen.

### Purification of Lipoproteins From Human Plasma

Plasma pools of normolipidemic donors were separated into total (TL), high-density (HDL), low-density (LDL) and very low-density (VLDL) lipoprotein fractions by flotation in a series of three centrifugations in which the density (ρ) of the plasma was progressively increased by adding powder KBr according to the procedure established by Havel et al. (1955) (Supplementary Figure S1A). The amount of KBr to be added was calculated using the Radding and Steinberg formula (Radding and Steinberg, 1960):

$${\text{X}}_{\text{KBr}}=\frac{V\times \text{(}\rho_{\text{f}}\text{-}\rho_{\text{i}})}{1-0.312\times \rho_{\text{f}}}$$

where:

X_KBr_ = amount of KBr in grams

V = volume of plasma sample in mL

ρ_f_ = final (desired) density to which the solution is to be adjusted

ρ_i_ = initial density of the solution to be adjusted

the numerical factor 0.312 = partial specific volume of KBr.

Briefly, plasma was divided into two aliquots, one of which was brought to ρ = 1.21g/mL, then centrifuged. The top fraction, containing TL, was collected while the bottom one discarded. The second plasma aliquot was centrifuged without addition of KBr. In this case, the top fraction, containing the VLDL, was stored while the bottom one was brought to ρ = 1.063 g/mL and centrifuged again. The top fraction of this second centrifugation, containing the LDL, was stored while the bottom one was brought to ρ = 1.21 g/mL and centrifuged again. The top fraction of this third centrifugation, containing the HDL, was stored while the bottom one was discarded. All centrifugations were performed at 40,000 rpm (Beckman L-70 ultracentrifuge, 60-TI rotor), at 8°C for 18 h. The fractions were made tissue culture grade by two sequential dialyses, one against H_2_O (to remove KBr) and the other one against Iscove Modified Dulbecco’s Medium (IMDM) (to balance their osmolarity), sterilized by filtration through a 0.45 μm filter and stored at -20°C. Possible lipid and protein losses induced by the dialysis and filtration processes were documented by determining the protein and lipid content of the tissue culture grade fractions. Quantitative analysis of the apolipoprotein composition of the individual lipoprotein fractions was performed by gel electrophoresis followed by Coomassie or Silver staining (Thermo Scientific, Rockford, IL, United States) (Supplementary Figure S1B) and densitometric scanning (Supplementary Figure S1C) (Chapman, 1986). Cholesterol and triglycerides were determined by commercially available enzymatic assays (cat. R1120 and R1373, BPC BioSed srl, Castelnuovo di Porto, Italy). The extent of loss was calculated by comparing the apoprotein/lipid enrichment ratio of each fraction with that reported in the literature (Supplementary Table S2) (Chapman, 1986). Although, as predicted, the composition of the tissue culture grade fractions is significantly different from that predicted by the literature, some specificity in protein and lipid composition was observed. In particular, Fraction 1 (i.e., purified VLDL) is rich in Apolipoprotein (Apo) B100, A1 and C1-3 and expresses a cholesterol/triglyceride ratio of 0.36. Fraction 2 (purified LDL) contains ApoB100 with traces of A1, A2, and C2-3, shows a protein/lipid ratio of 4.5 and contains approximately three times more cholesterol than triglycerides (cholesterol/triglycerides ratios = 2.83). Fraction 3 (purified HDL) contains ApoB100, A4, A1, A2, C1 and traces of E and human serum albumin (HSA). The cholesterol/triglycerid ratio of this fraction is similar to that of Fraction 2 (2.5 vs. 2.8).

### *Ex vivo* Expansion of Human Erythroblasts

MNC (10^6^ cells/mL) were cultured under human erythroid massive amplification (HEMA) conditions in the presence of human SCF (10 ng/mL, Amgen, Thousand Oaks, CA, United States), EPO (3 U/mL, Janssen, Raritan, NJ, United States), IL-3 (1 ng/mL, PeproTech, Rocky Hill, NJ, United States), Dex and estradiol (both 10^-6^M, Sigma Aldrich, Saint Luis, MO, United States). The culture media was represented by IMDM supplemented with either FBS (HEMA^ser^) (Migliaccio et al., 2002), or clinical grade components mostly of human origin (HEMA^def^) (Migliaccio et al., 2010) (Supplementary Figure S2). The components of HEMA^def^ are deionized HSA (10% v/v, Baxter International Inc., Deerfield, IL, United States), human iron saturated transferrin (TRF), recombinant human insulin (Merck KGaA, Darmstadt, Germany), β-mercaptoethanol, sodium pyruvate, nucleosides, trace elements and L-glutamine (all from Sigma-Aldrich). Sources of lipids were represented by either home-made (HM) liposomes composed by HSA, cholesterol (400 μg/mL, Cat. No. C3045, Sigma-Aldrich) and soybean lecithin (1.2 mg/mL, Cat. No. P3644, Sigma-Aldrich), or culture-grade lipids from commercial sources or lipoprotein fractions purified from human plasma described above. The commercial lipids investigated were: A: Lipids Cholesterol Rich from adult bovine serum (Cat. L-4646); B: Fatty Acid Supplement (Cat F7050); C: Lipid Mixture (Cat. L-0288) (all from Sigma-Aldrich), D: Chemically defined Lipid Concentrate (Cat. 11905-031, Gibco Invitrogen, Carlsbad, CA, United States) and E: commercial tissue culture grade LDL (Cat. LP2-2MG, Sigma). LDL-Sigma contain more lipids (78–80% vs. 18%) but less proteins (22–20% vs. 82%) than HM-LDL (Supplementary Table S3). Cultures were incubated at 37°C in a fully humidified 5% CO_2_ atmosphere and the cells analyzed every 2–4 days up to days 18 of culture. A list of the end-points analyzed in this study and their biological implications is provided in Supplementary Table S1.

### Determinations of Cell Numbers, Viability and Phenotype

Cell numbers and viability were determined by microscopic evaluation of cells stained with trypan blue (Boston Bioproducts, Ashland, MA, United States) in a Burker chamber. The maturation state was assessed by flow cytometry on the basis of CD235a (glycophorin A) and CD36 (the thrombospondin receptor) expression using the phycoerythrin (PE)–conjugated CD36 and allophycocyanin (APC)-conjugated CD235a antibodies, or appropriate isotype controls (all from BD-Pharmigen, San Diego, CA, United States) (Migliaccio et al., 2011). Maturation stages were confirmed by visual examination of cytocentrifuged smears (Cytospin 3, Shandon, Astmoor, United Kingdom) (Chapman, 1986). Dead cells were excluded by Sytox Blue staining (0.002 mM, Molecular Probes). Fluorescence was measured with a FACS Aria and data were analyzed with the FlowJo software (Tree Star, Inc., Ashland, OR, United States). Sensitivity to autophagic death was determined by Acridine Orange (AO) staining (Fluka Biochemika, Buchs, Switzerland), as described (Migliaccio et al., 2011).

### Colony Forming Assay

MNC (10^5^ cells/plate) were cultured in semisolid media under conditions resembling either HEMA^ser^ or HEMA^def^ (cf. previous sections). HEMA^ser^ cultures were represented by a commercial semisolid methylcellulose assay containing FBS (30% v/v, MethoCult Stem Cell Technology, Inc., Vancouver, BC, Canada). HEMA^def^ cultures were represented by a home-made methylcellulose assay in which FBS is replaced by deionized human serum albumin (HSA) and HSA-adsorbed cholesterol (final concentrations, 2 × 10^-4^mol/L), iron-saturated transferrin (5 × 10^-7^mol/L), insulin (1.7 × 10^-6^mol/L), nucleosides (10 μg/mL each), sodium pyruvate (10^-6^mol/L) and L-glutamine (2 × 10^-3^mol/L), as previously described (Migliaccio and Migliaccio, 1987). All the cultures were stimulated with human SCF (100 ng/mL), IL-3 (10 ng/mL), GM-CSF (10 ng/mL), G-CSF (100 ng/mL) and EPO (5 U/ml), as described (Masiello et al., 2013). Plates were incubated at 37°C in a fully humidified incubator containing 5% CO_2_. Colonies were scored after 8 (colony forming unit-granulocytic erythroid, CFU-E) and 14 (burst forming unit-erythroid, BFU-E, colony forming unit-granulo-monocytic, CFU-GM and colony forming unit-granulocyte, eythroid, megakaryocyte and monocyte, CFU-GEMM) days according to standard morphological criteria (Masiello et al., 2013).

### Proliferation Assay

Cell proliferation was assessed by the 3-(4,5-dimethylthiazol-2-yl)-2,5-diphenyltetrazolium bromide (MTT) assay as described (Falchi et al., 2015) where the MTT assay was validated as an index of erythroblast proliferation by careful correlations between MTT values and increased cells numbers detected by visual cell counting. Briefly erythroblasts (5 × 10^5^cells/100 μL/well) were plated in triplicate wells of a 96-well microtitre flat-bottomed plate in the absence or presence of increasing concentrations of commercially available lipid supplements or individual lipoprotein fractions and cultured for 72 h at 37°C in a humidified incubator with 5% CO_2_ in air. Ten μL of MTT [5mg/mL] were then added to each well and incubated at 37°C in a humidified atmosphere for additional 4hrs. The solution was removed and the resulting formazan salts dissolved with Sorensen’s Glycine Buffer (0.1M Glycine plus 0.1M NaCl in PBS). Optical densities were measured at 540 nm in the VICTOR3^TM^ microplate reader (Perkin Elmer, Waltham, MA, United States) and expressed as absorbance (ABS) values.

### Expression Profiling by Microarray Analyses

The expression profile of human erythroblasts generated at days 10 in HEMA^ser^ with or without Dex was previously published (Hricik et al., 2013). The list of differentially expressed genes was manually searched for those involved in lipid metabolism.

### Statistical Analysis

Results are presented as Mean (±SD) of at least three separate experiments, unless stated otherwise. Statistical analysis was performed by paired *t*-test and ANOVA (Origin 6.0 for Windows, Microcal Software, Inc., Northampton, MA, United States) and considered statistically different with a *p* < 0.05.

## Results

### The Expression Profiling of Erythroblasts Generated in the Presence of Dex Predicts Altered Lipid Metabolism

Given the great role played by glucocorticoids in the development of fatty liver disease (Woods et al., 2015), most of what is known on the effects of these hormones on lipid metabolism has been obtained in liver using holistic experimental models. These studies have indicated that glucocorticoids promote the availability of cholesterol indirectly, by increasing the production of VLDL by liver cells, and that of triglycerids directly, by promoting their *ex novo* synthesis by the cells (Bagdade et al., 1976). By contrast, there is little information on the role played by glucocorticoids in the control of lipid metabolism in erythroid cells.

To clarify the genes targeted by Dex in erythroid cells, in a previous study we compared the expression profiling of erythroblasts generated by days 10 in HEMA^ser^ with and without Dex (Hricik et al., 2013). We chose days 10 because this time point contained great numbers of erythroblasts in the process of switching from a proliferation to a maturation mode (Migliaccio et al., 2011), allowing us to detect targets affecting both processes. This study has revealed that erythroblasts generated with and without Dex differ in the expression of only 55 genes (Hricik et al., 2013). By manual analyses, we discovered that six of the differentially expressed genes (approximately 10%) are involved in lipid metabolism, three of which at the mitochondria level (Table 1).

**Table 1:** Genes encoding proteins controlling lipid metabolism that are differentially expressed on erythroid cells obtained from adult sources in cultures stimulated with and without Dex.

		Gene symbol	Fold Change	*p*-value	Name	Function
Effects of Dex	Activation	HMGCL	+1.55	2.15E-0.5	3-Hydroxymethyl-3-Methylglutaryl-CoA Lyase	Mitochondrial enzyme involved in the ketogenic pathway
		ABCA1	+2.18	1.71E-0.5	ATP-binding cassette, sub-family A, member 1	Mediator of cholesterol efflux
		HSD17B7	+1.49	1.55E-0.5	Hydroxysteroid 17-Beta Dehydrogenase 7	Postsqualene Enzyme of Cholesterol Biosynthesis
	Inhibition	OXCT1	-1.51	4.50E-0.5	3-Oxoacid CoA-Transferase 1	Mitochondrial enzyme involved in the ketogenic pathway
		GPAM	-1.38	2.03E-0.5	Glycerol-3 Phosphate Acyltransferase, Mitochondrial	Rate limiting enzyme of triacylglycerol biosynthesis
		PRKACB	-1.14	6.19E-0.5	Protein Kinase CAMP-Activated Catalytic Subunit Beta	Mediator of cAMP signaling in several metabolic processes, including in the regulation of lipid metabolism

*Symbols of the genes activated (positive fold change) and inhibited (negative fold change) by Dex.*

Dex activates and inhibits, respectively, the expression of HMGCL and OXCT1, which are synergistically involved in the terminal stages of lipid degradation and in the ketogenic pathway in mitochondria (Fu et al., 2010; Fukao et al., 2014), and increases the expression of ABCA1, which encodes a cholesterol efflux pump (Litvinov et al., 2018). Dex also activates HSD17B7, which encodes an enzyme of the cholesterol biosynthetic pathway (Marijanovic et al., 2003), and decreases the expression of PRKACB, the signaling mediator of several metabolic processes, and of GPAM, the gene that codes for rate-limiting enzyme (GPAT1) of the glycerolipid biosynthetic pathway (Wendel et al., 2009; Søberg and Skålhegg, 2018).

These results suggest that Dex impairs the overall cell metabolism, including lipid metabolism, making the membrane homeostasis of these cells, and possibly additional functions, particularly dependent on lipids up taken from exogenous sources.

### Numerous Erythroblasts Generated in the Presence of Dex Present Morphological Evidence of Plasma Membrane and Mitochondria Fragility

**FIGURE 1:**
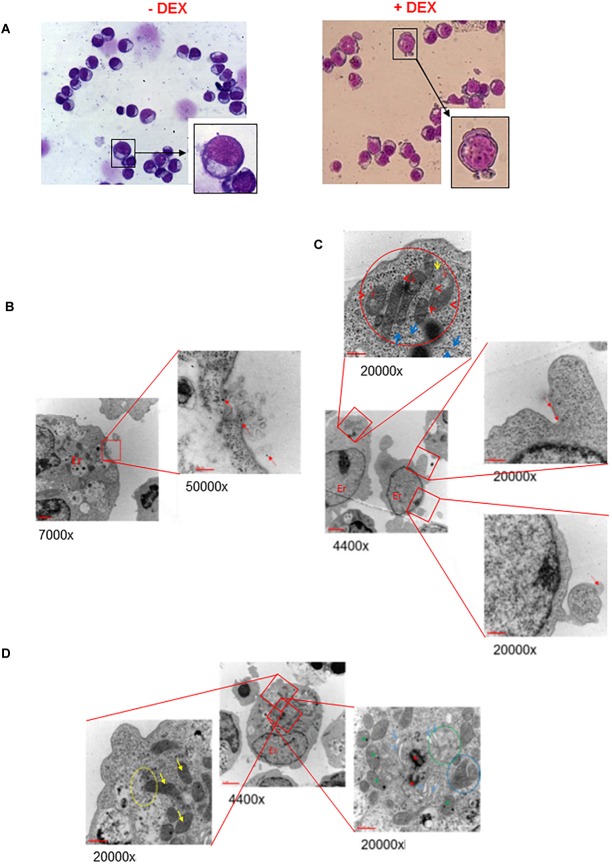
*Ex vivo* generated erythroblasts present morphological evidence of plasma and mitochondrial membrane damage. **(A)** The erythroblasts plasma membrane presents numerous pebbles. May-Grunwald-staining of representative cytospin preparations of erythroblasts from days 10 HEMA^def^ culture obtained with and without Dex, as indicated. Representative pro-erythroblasts are shown at higher magnifications in the inserts. Original magnification 40X. **(B,C)** The membrane pebbles present ultrastructural features associated with exosome and micro-particle release. Electron microscopy analyses of the pebbles on the plasma membrane of representative pro-erythroblasts from days 10 HEMA^def^ with Dex. **(B)** At greater magnification, the pebble, probably a multivesicular body, shows eruption of the plasma membrane with extrusion of exosomes and free ribosomes (red arrows). **(C)** At greater magnification, the pebble shows the presence of membrane leaking (red arrows) and release of micro-particles (panel of the bottom). Greater magnification of this pro-erythroblast (panel on the top) also shows the localization of the mitochondria at a pole of the cytoplasm, the mitochondria body. In addition, the mitochondria present morphological sign of distress (red circle) while the cisterns of the surrounding rough endoplasmic reticulum present numerous traits deprived of ribosomes (blue arrows). **(D)** The mitochondria from erythroblasts cultured with Dex present several abnormalities of the crest membranes. Electron microscopic analyses of a representative pro-erythroblast showing at greater magnification that the mitochondria present several crest abnormalities (reduction in number, diameter larger than normal and membrane ruptures). Only the most external mitochondria (yellow circle) appears partially fused with a phagosome (yellow arrow, bottom left panel). The panel on the right shows fusion among mitochondria (blue circle). Red asterisks indicate centrioles and green asterisks autophagy vesicles. Original magnifications are indicated below each panel.

To assess whether possible impairment of lipid metabolism may affect the quality of the plasma membrane, we first compared by optical microscopy the morphology of erythroblasts generated by days 10 in the presence and absence of Dex (Figure 1A and results not shown). These observations revealed that cultures with Dex contain greater numbers of erythroid cells presenting alterations of their membrane resembling protrusions or pebbles than those without Dex. In the case the pro-erythroblasts, easily recognizable on smears by their typical morphology and larger size (>30 μm) (Insert in Figure 1A), the frequency of cells presenting pebbles was 14 ± 5% in HEMA^ser^ and 23 ± 7% in HEMA^def^ (*p* < 0.05), respectively.

The fine structure of the membrane abnormalities was defined by electron microscopy analysis of erythroblasts expanded in HEMA^def^, a condition considered superior for generating cRBCs because it sustains levels of amplification 10-times greater than HEMA^ser^ (Migliaccio et al., 2010). Also this analysis was focused on cells with the ultrastructural characteristics of pro-erythroblasts obtained at days 10 of culture. These studies provided evidence that the membrane pebbles are associated with two different types of ultrastructural abnormalities. The first abnormality resembles a burst bubble of plasma membrane with leakage of intracytoplasmic material, as well as of exosomes (<20 nm in diameter) in the environment, a clear indication of membrane fragility, that possibly originated from nearby multivesicular bodies (Raposo and Stoorvogel, 2013) (Figure 1B). The second abnormality resembles micro-vesicles ranging in diameter from 105 to 3500 nm (with an average of 1410 ± 877 nm) being released in the environment (Figure 1C).

These results indicate that pro-erythroblasts generated with Dex are prone to express membrane abnormalities leading to exosome and microvesicle release, a feature that in RBCs has been reported associated with conditions of cellular distress (Raposo and Stoorvogel, 2013; Leal et al., 2018).

Electron microscopy analysis also allowed us to monitor the plasma membrane of the mitochondria (Figure 1C,D). In pro-erythroblasts, mitochondria are localized at a pole of the cytoplasm forming a structure that we define mitochondrial body (top panel in Figure 1C). Most, if not all, of the mitochondria are abnormal in morphology with reduced and enlarged internal crests (top panel in Figure 1C,D). Only a minority of them, mostly those located in the periphery of the mitochondrial body, appears engulfed into autophagic vesiscles in preparation of the mitophagic degradation that leads to formation of reticulocytes (Moras et al., 2017).

These results provide morphological evidence suggesting that, during erythroid maturation, mitochondria lose their metabolic functions, including those involved in lipid biosynthesis, at the pro-erythroblast stage before the formation of the mitophagic vesicles required for terminal maturation and that these processes may be affected by Dex.

### Commercial Lipid Supplements and Lipoproteins Purified From Human Plasma Improve Short-Term Proliferation of Human Erythroblasts in Culture

The morphological data presented above (Figure 1) suggest that the plasma membrane abnormalities of erythroblasts generated under current culture conditions are possibly influenced by insufficient and/or not appropriate lipid supplementation in the culture media.

**FIGURE 2:**
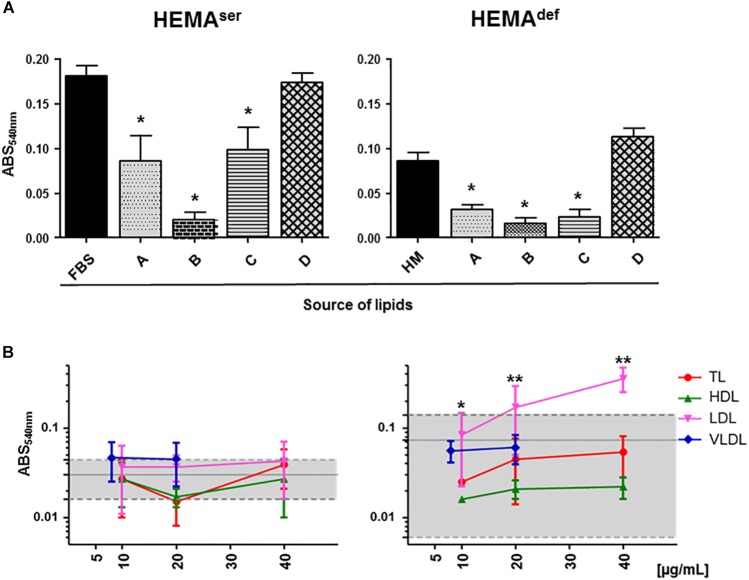
Plasma lipoproteins increase short-term proliferation of human erythroblasts in HEMA^def^. **(A)** MTT proliferation assay of days 6 erythroblasts cultured for 4 days under HEMA^ser^ (left panel) or HEMA^def^ (right panel) conditions in the presence of commercially available lipid preparations. Source of lipids: **(A)** Lipids Cholesterol Rich from adult bovine serum (Cat. L-4646, Sigma-Aldrich); **(B)** Fatty Acid Supplement (Cat. F7050, Sigma-Aldrich); **(C)** Lipid Mixture (Cat. L-0288, Sigma-Aldrich) and **(D)** Chemically defined Lipid Concentrate (Cat. 11905, Gibco). In control cultures, the source of lipids was FBS (HEMA^ser^) or home-made liposomes (HM, HEMA^def^) designed to achieve maximal erythroblast amplification in chemically defined culture conditions (31) (see Materials and Methods for further details). Results are expressed as absorbance (ABS) at 540 nm and are presented as Mean (±SD) of three experiments performed in triplicate, each one with a different donor. Results statistically different from the corresponding controls (*p* ≤ 0.01, by paired *t* test) are indicated by ^∗^. **(B)** MTT proliferation assay of days 6 erythroblasts cultured for 4 days in the presence of increasing concentrations of lipoproteins purified from human plasma as described in Supplementary Figure S1. Controls were represented either by FBS (HEMA^ser^) or home-made liposomes (HEMA^def^) and are indicated by the gray areas. Results are presented as Mean (±SD) of three experiments performed in triplicate. In HEMA^def^, results obtained with LDL are statistically different from the corresponding controls with *p* ≤ 0.05 (^∗^) at 10 μg/mL and *p* ≤ 0.01 (^∗∗^) at 20 and 40 μg/mL (by paired *t* test).

To begin assessing the role exerted by lipid supplementation on *ex vivo* erythroblast expansion, we first determined the level of short-term proliferation of these cells under HEMA^ser^ and HEMA^def^ conditions supplemented either with lipid formulations commercially available or with lipoprotein fractions purified from human plasma (Figure 2).

Both under HEMA^ser^ and HEMA^def^ conditions, commercially available supplements sustain levels of proliferation similar (chemically defined lipid concentrate from Gibco), or greatly inferior (see the toxicity exerted by the Fatty Acid Supplement cat L-0288 from Sigma), to those observed in controls (Figure 2A). By contrast, all the lipoprotein fractions (regardless of the concentration used) sustain levels of erythroblast proliferation similar to controls in HEMA^ser^, which already contain bovine lipoproteins provided by FBS (Figure 2B). Of note, TL, HDL and VLDL sustain erythroblast proliferation similar to control in HEMA^def^, that does not contain any additional lipid supplement while LDL significantly increases erythroblast proliferation with respect to controls in a concentration dependent fashion with maximal effects observed at 20–40 μg/mL.

These results indicate that while none of the commercially available lipid supplements is capable to improve proliferation of human erythroblasts above controls, the lipoproteins purified from human plasma, and in particular LDL, which is the fraction with the greatest protein content (Supplementary Table S2), may represent a better source of lipids than HM liposomes in HEMA^def^.

### LDL Increases the Number of BFU-E-Derived Colonies Generated in Semisolid Media Under Both HEMA^def^ and HEMA^def^ Conditions

To start assessing the effect of lipid supplementation on erythroid differentiation, we first enumerated the number of hematopoietic colonies generated in semisolid assay by MNC from normal blood donors under HEMA^ser^ and HEMA^def^ conditions supplemented with lipoproteins purified from human plasma (Table 2). In these, as well in all the other experiments described from now on, lipoprotein fractions were tested at a concentration of 20 μg/mL. These experiments tested also commercially available LDL for comparison.

**Table 2:** Effects of lipoproteins purified from human plasma on the number of colonies generated by human MNC in semisolid cultures under HEMA^ser^ and HEMA^def^ conditions.

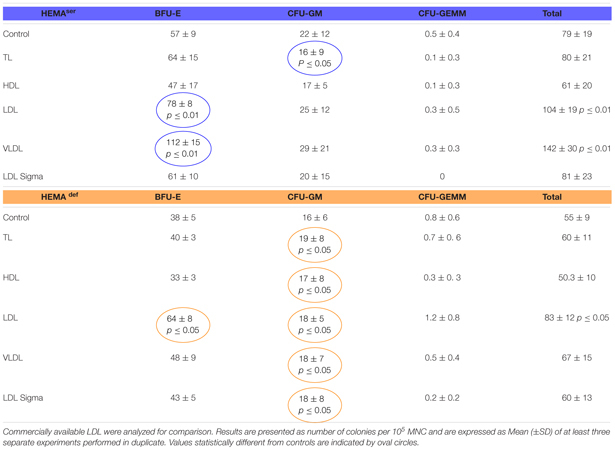

Both TL in HEMA^ser^ and all the lipoprotein fractions in HEMA^def^ have modest, although significant, effects on the growth of CFU-GM-derived colonies while do not improve the growth of GFU-GEMM-derived colonies (Table 2). By contrast, LDL (by 30%) and VLDL (by approximately 100%) significantly increase the numbers of BFU-E-derived colonies generated in HEMA^ser^ (78 ± 8 and 112 ± 15 vs. 57 ± 9, *p* < 0.01) while LDL increase (by 1.5-fold) that of BFU-E-derived colonies in HEMA^def^ (64 ± 8 vs. 38 ± 5, *p* < 0.05). To be noted that the commercial LDL does not improve colony growth neither in HEMA^ser^ not in HEMA^def^.

These results indicate that the LDL (and to some extent VLDL) fraction purified from human plasma increases the number of BFU-E induced to form erythroid colonies in semisolid cultures.

### Both LDL and VLDL Increased the Number of Erythroblasts Generated in Liquid Culture by Adult MNC Under HEMA^def^ Conditions

**FIGURE 3:**
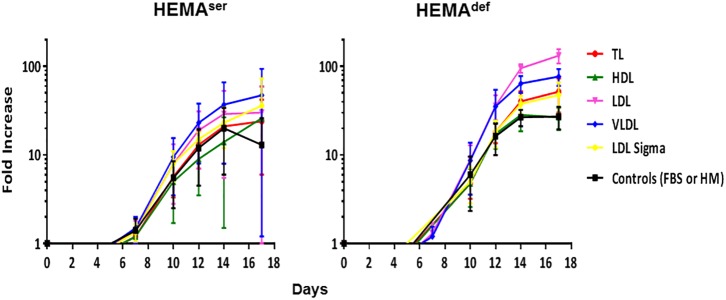
LDL and VLDL significantly increase the numbers of erythroblasts generated in HEMA^def^. Total number of cells, expressed as Fold Increase (FI), generated over time in HEMA^ser^ **(left)** and HEMA^def^ **(right)** supplemented with lipoproteins purified from human plasma (TL, red; HDL, green; LDL, pink; VLDL, blue, all at 20 μg/mL). Results are compared with those obtained with FBS alone (HEMA^ser^), home-made liposomes (HEMA^def^) (controls, black) or LDL from a commercial source (Cat. L-0288 from Sigma, yellow). Results are presented as Mean (±SD) of four experiments each one with a different donor. In HEMA^ser^, the experimental points are not statistically different from controls. In HEMA^def^, LDL at days 14 (*p* = 0.008) and days 17 (*p* = 0.013) and VLDL at days 14 (*p* = 0.004) and days 17 (*p* = 0.029) are statistically different from the corresponding controls. Commercial LDL is statically different only at days 3 (*p* = 0.012).

To further explore in some detail the effects exerted by the lipoprotein fractions, we also evaluated the levels of erythroblast expansion in liquid culture supplemented with these preparations (Figure 3). In HEMA^ser^, the various lipoprotein fractions investigated do not improve the overall fold increase (fold-increase ∼20–40 by days 14–17 in the presence of all supplements). By contrast, both LDL and VLDL increase by 3-fold the total number of erythroblasts generated by days 14–17 under HEMA^def^ conditions (Fold Increase 95 ± 10 and 64 ± 14 vs. 27 ± 6, *p* < 0.01–0.05, respectively for LDL and VLDL compared to control value at the same time point) (Figure 3 and Table 3). On note, the commercial LDL generates erythroblasts in numbers similar to controls.

**Table 3:** Summary of the effects induced by the various lipoprotein fractions on the end-points used to assess the efficiency of erythroid expansion.

	Erythroid Expansion (Day 14)		Progenitor recruitment (Day 0)		Progenitor expansion (Day 10)		Erythroblast proliferation (Day 10)	
	Fold Increase		BFU-E/10^5^ MNC^∗∗^		Tot CFU/Culture (x10^3^)		ABS 540nm (x10^2^)	
	HEMA^ser^	HEMA^def^	HEMA^ser^	HEMA^def^	HEMA^ser^	HEMA^def^	HEMA^ser^	HEMA^def^
Control	20 ± 14	27 ± 6	57 ± 9	38 ± 5	n.d.	542 ± 54	3.0 ± 1.4	7.3 ± 6.7
TL	21 ± 13	40 ± 12	64 ± 15	40 ± 3	n.d.	764 ± 216	1.5 ± 0.7	4.5 ± 3.2
HDL	14 ± 12	28 ± 10	47 ± 17	33 ± 3	n.d.	620 ± 100	1.7 ± 0.4	2.1 ± 0.5
LDL	29 ± 23	95 ± 10 *p* ≤ 0.01	78 + 8 *p* < 0.01	64 + 8 *p* < 0.05	n.d.	756 ± 104 *p* ≤ 0.01	3.7 ± 1.2	17.1 ± 12.1 *p* ≤ 0.05
VLDL	37 ± 29	64 ± 14 *p* < 0.05	112 ± 15 p < 0.01	48 ± 9	n.d.	1064 ± 292 *p* ≤ 0.05	4.5 ± 1.3	6.1 ± 2.2
LDL Sigma	23 ± 11	38 ± 9	61 ± 10	43 ± 5	n.d.	680 ± 84	n.d.	7.9 ± 4.4

**FIGURE 4:**
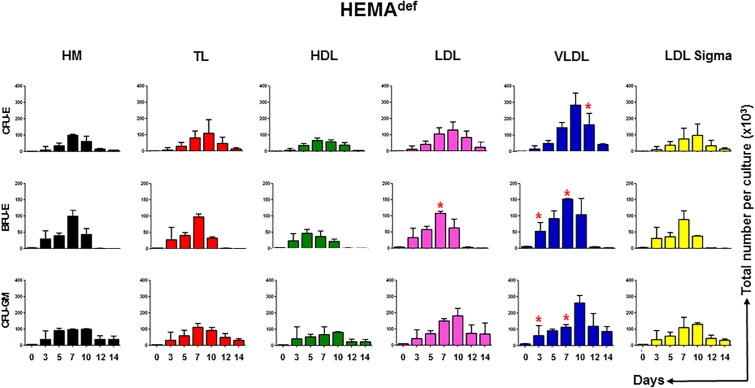
LDL and VLDL increase the expansion of erythroid progenitors under HEMA^def^ conditions. Number of progenitor cells able to generate erythroid (BFU-E and CFU-E) and myeloid (CFU-GM) colonies present over time in HEMA^def^ supplemented with either home-made liposomes (HM) or with TL, HDL, LDL or VLDL (all at 20 μg/mL). Results are expressed as number of progenitor cells per culture and are presented as Mean (±SD) of four experiments each one with a different donor. See legend of Figure 2 for further detail. Values statistically different (*p* < 0.05 by paired *t*-test) with respect to the corresponding data obtained with HM are indicated by ^∗^. Values obtained with LDL-Sigma are not statistically different neither from those obtained with HM nor from those observed with LDL.

*Results are presented as Mean (±SD) of three experiments performed in triplicate. Experiments were performed in parallel analyzing the same donor under HEMA^ser^ and HEMA^def^ conditions (three separate donors per experimental point). Total colony forming units, CFU total number of colonies generated in semisolid cultures (see Table 2 and Figure 2–4, 6 for further detail).*

### Both LDL and VLDL Increase the Expansion of Erythroid Progenitors in HEMA^def^

To clarify the mechanisms underlying the increased erythroblast expansion observed in HEMA^def^ supplemented with LDL and VLDL, we evaluated the effect exerted over time by the various lipoprotein supplements on the number of hematopoietic progenitor cells in liquid culture. Hematopoietic progenitor cells were determined by culturing aliquots harvested from the liquid cultures at days 3, 5, 7, 10, and 14 under semisolid conditions and then scoring the number and type of colonies generated by these cell aliquots (Figure 4).

In cultures supplemented with HM liposomes, the number of BFU-E and CFU-E progressively increases and then declines reaching the maximum by days 7. In these cultures, BFU-E are barely detectable by days 12. In cultures supplemented with TL and HDL and with commercial LDL, the number of erythroid progenitors increases over time with amplitude and kinetics similar to that observed in controls. By contrast, in cultures supplemented with LDL and VLDL, the maximal levels of amplification of progenitor cells of all types is significantly greater than in controls and the numbers of CFU-E (and CFU-GM) reach their peak at days 10 instead than at days 7.

These results suggest that improved and prolonged expansion of hematopoietic progenitor cells contributes to the greater numbers of erythroblasts observed in HEMA^def^ supplemented with LDL and VLDL.

### Erythroblasts Generated in the Presence of VLDL Are Resistant to Death by Autophagy Induced by Growth Factor Deprivation (GFD)

One of the factors limiting erythroblast expansion in HEMA is represented by the great number of cells that die by autophagy at the end stage of the culture (Migliaccio et al., 2011). To further investigate the mechanism that underlay the improved erythroblast expansion observed in the presence of selected lipid supplements, we assessed the susceptibility to death of erythroblasts generated *ex vivo* in HEMA^def^ supplemented with HM liposomes or with VLDL. Sensitivity to death was determined using as surrogate marker the frequency of erythroblasts undergoing autophagy [identified by Acrydin Orange (AO) staining] following 4 h of GFD, as described (Migliaccio et al., 2011).

**FIGURE 5:**
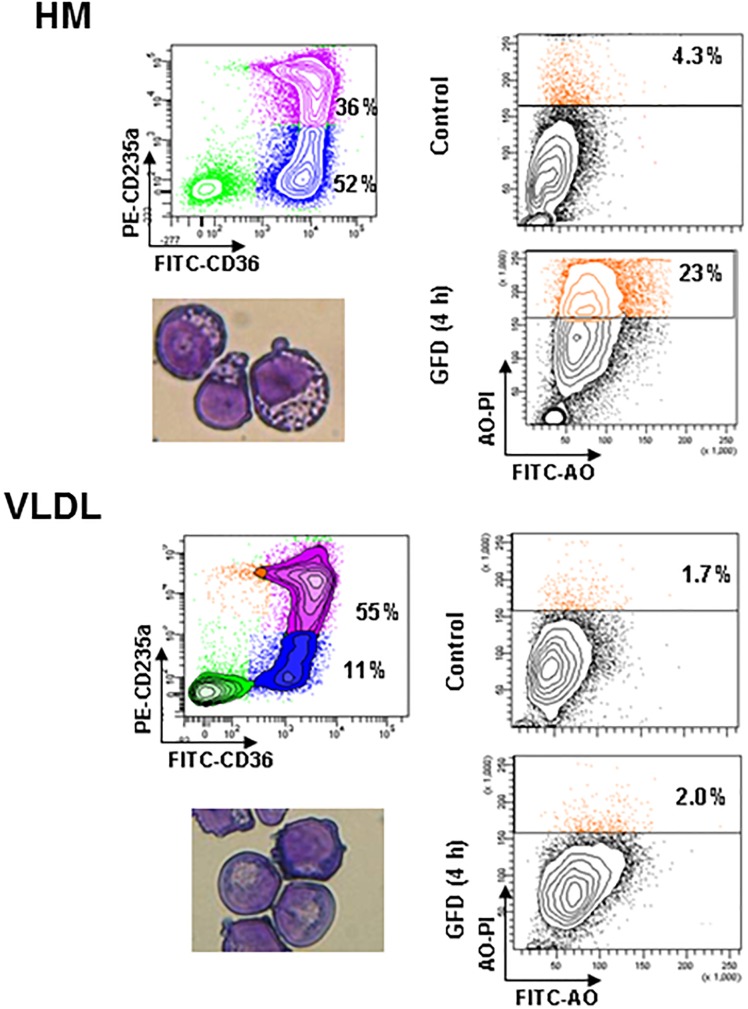
Erythroblasts generated in the presence of VLDL are resistant to autophagic death induced by growth factor deprivation. The panels on the left present the flow cytometry and morphological analyses of erythroblasts generated by days 10 in HEMA^def^ supplemented either with HM liposomes or with VLDL (20 μL/mL). The panels on the right compare the frequency of Acrydine Orange (AO) positive cells, as indication of autophagic death, of the two populations when cultured for 4 h with (Control) or without growth factors (growth factor deprived, GFD). Results are representative of those obtained in two separate experiments.

A great number (23%) of erythroblasts generated in the presence of HM liposomes became positive to AO staining after GFD (Figure 5). By contrast, only 2% of erythroblasts generated with VLDL became AO positive after GFD, an indication that they are resistant to GFD-induced death. Interestingly, the cytoplasm of erythroblasts generated with VLDL also contain fewer vacuoles and their plasma membranes contain less pebbles than erythroblasts generated with HM (14 ± 6 vs. 23 ± 7, *p* < 0.05) (see also the morphological analyses presented in Figure 5).

### All the Lipoprotein Fractions From Human Plasma Increase the Level of Terminal Erythroid Maturation Observed by Days 17 in HEMA^def^

**FIGURE 6:**
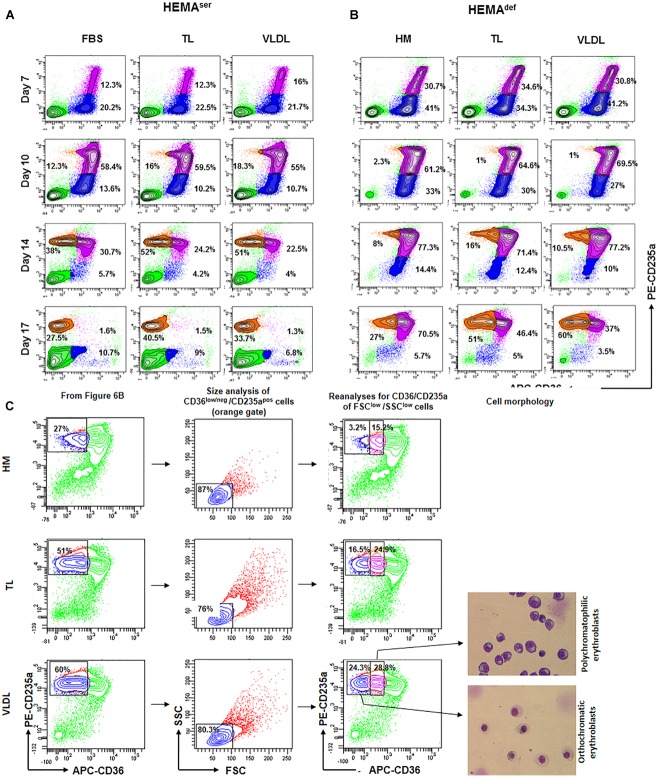
All the lipoprotein fractions increase terminal erythroid maturation under HEMA^def^ conditions. Contour plots for CD36 and CD235a expression obtained by FACS of erythroblasts generated over time in HEMA^ser^ **(A)** and HEMA^def^ **(B)** supplemented with TL and VLDL. Cells obtained with FBS or with HM liposomes were analyzed as control. Similar results were observed in cultures supplemented with HDL, LDL and LDL-Sigma (results not shown). Analyses for CD36/CD235a expression identifies five populations corresponding respectively to lymphocytes (CD36^neg^CD235a^neg^ cells, bright green); pro-erythroblasts (CD36^pos^CD235a^neg^ cells, blue); basophilic-erythroblasts (CD36^pos^CD235a^high^ cells, purple); and polychromatophilic/orthochromatic (CD36^neg^CD235a^high^ cells, orange) erythroblasts. The frequencies of pro-erythroblasts, basophilic and poly/orthochromatic erythroblasts are indicated within the quadrants. Results are representative of those obtained in two separate experiments. **(C)** Contour plot for forward scatter (FSC) and side scatter (SSC) of the days 17 cells in the CD36^low/neg^/CD235a^pos^ gate of Figure 6B. The cells were then re-analyzed for CD36/CD235a expression, sorted and stained with May-Grunwald Gimsa. The numbers inside the panels indicate the frequency of the cells in the different gates. This analysis allows discriminating between erythroid cells at the polychromatophilic and orthochromatic stage. Only results obtained in the HM, TL, and VLDL group are presented. The results obtained with TL and VLDL are representative of those obtained with HDL, LDL, and LDL-Sigma.

In spite of the presence of Dex, erythroblasts in the proliferation phase eventually progress to terminal maturation. Therefore, to assess the effects of lipid supplementation on the efficiency of terminal erythroid maturation, the maturation profile expressed by erythroblasts generated over time under HEMA^ser^ and HEMA^def^ supplemented with the various lipoprotein fractions was assessed (Figure 6). These studies exploited the fact that profiling for CD36/CD235a expression divides human erythroblasts into three maturation classes corresponding respectively to pro-erythroblasts (CD36^pos^CD235a^neg^ cells); basophilic-erythroblasts (CD36^pos^CD235a^high^ cells); and poly-chromatophilic/orthochromatic erythroblasts (CD36^neg^CD235a^high^ cells) (Falchi et al., 2015). The transition from basophilic- to ortho-erythroblasts is also associated with a dramatic reduction in size (from 24 to 19 μm) recognizable by forward/side scatter analyses.

According to our results, in HEMA^ser^, the cells progress along the maturation pathway with the same kinetics irrespective of the lipids used as supplement (Figure 6A and results not shown). Mature erythroblasts (CD36^pos^CD235a^high^) represent approximately 12–16% of the population by days 7 while all the erythroblasts express the mature CD36^neg^CD235a^high^ phenotype by days 17. By days 17, the frequency of non-erythroid cells in HEMA^ser^ is high (46%). Since non-erythroid cells do not proliferate in HEMA (Migliaccio et al., 2002), the relative increase of this population over time is a further indication of the great rates of death of erythroblasts at the end stage of HEMA (Migliaccio et al., 2011). The addition to HEMA^ser^ of any of the lipoprotein fractions tested in this study does not affect the kinetics of the maturation process but increases the frequency of mature erythroblasts, which reaches 30-50% by days 17, an indication that the presence of lipoproteins increases survival of mature erythroblasts.

The kinetics of erythroblast maturation in HEMA^def^ is visible different than that observed in HEMA^ser^ (Figure 6B and results not shown). In control cultures, immature CD36^pos^CD235a^neg^ erythroblasts are detected with a frequency (30%) significantly greater than that observed in HEMA^ser^ (12%) by days 7 and the majority of the cells remains immature until days 17 when this population still represents 70% of the cells. Conversely, by days 17 mature erythroblasts represent only 27% of the cells. A great difference between HEMA^ser^ and HEMA^def^ is represented by the frequency of non-erythroid cells which are barely detectable in HEMA^def^, an indication of better erythroblast survival. Of note, the replacement of the HM liposomes with the lipoprotein fractions does not affect the kinetics of erythroblast maturation but doubles the frequency of mature erythroblasts detected in HEMA^def^ by days 17 (40–60% vs. 27%) (Figure 6B and results not shown).

To further define the maturation stage of erythroid cells obtained by days 17 in HEMA^def^ supplemented with HM, TL and VLDL, the cells in the CD36^low^/CD235a^high^ gate were reanalyzed for forward scatter (FSC) and side scatter (SSC), two parameters which provide indication for size and cytoplasm granularity. The events in FSC^low^/SSC^low^ gate were then re-analyzed for CD36/CD235a expression and sorted for morphological analyses by May-Grunwald staining (Figure 6C). These analyses indicate that in all the experimental groups the mature cells are small in size. However, the mature gate in the HM group contains mostly polychromatophilic erythroblasts while those in the TL and VLDL groups contain both polychromatophilic and orthochromatic erythroblasts in a 50:50 ratio, these results indicate that lipoproteins improve terminal erythroidmaturation in HEMA^def^.

## Discussion

The conditions used to generate cRBCs for the first-in-man transfusion, as HEMA^ser^, contain FBS (Giarratana et al., 2005) and are not suited to produce cRBCs for multiple transfusions because it can be predicted that, as observed with other cellular products produced with FBS (Selvaggi et al., 1997; Tuschong et al., 2002), will trigger immunological reactions against bovine proteins when transfused into patients. Therefore, the formulation of clinical grade conditions depleted of FBS represents one of the major challenges in designing the manufacturing process of cRBCs (Migliaccio et al., 2012). To address this challenge, we formulated HEMA^def^, a media in which FBS is replaced with clinical grade HSA that sustains erythroblast expansion in proliferation cultures at levels 10-fold greater than those observed with HEMA^ser^ (Migliaccio et al., 2010). However, an important drawback of HEMA^def^ is represented by the source of lipids which is represented by HM liposomes generated by sonicating clinical grade HSA with egg-derived cholesterol and soybean lecithin. Given the known intolerance to both products existing in the human population, HEMA^def^ may not be indicated to produce cRBCs for the general population.

To formulate clinically more suited culture conditions, we determined *ex vivo* expansion of erythroblasts in HEMA^def^ formulated either with commercial lipid supplements or with HM lipoprotein fractions purified from human plasma. Parallel experiments were conducted in HEMA^ser^ for comparison. Our results demonstrate that all the commercially available lipid supplements tested in this study, including LDL from Sigma, generated erythroblasts in numbers inferior to those sustained by HM liposomes (Figure 2). By contrast, supplementation of HEMA^def^ with either LDL or VLDL purified in house from fresh human plasma supports the generation of erythroblasts in numbers significantly greater (2–3-times) than those generated by HM liposomes (Table 3).

LDL and VLDL fractions increase the numbers of erythroblasts generated in culture through mechanisms targeting both the erythroid progenitor and precursor cells (Table 3). At the progenitor level, they significantly increase the number of cells recruited to mature (Table 2) and their expansion in liquid culture (Figure 4). At the precursor level, they increase proliferation rates (Figure 2) and resistance to autophagic death (Figure 5). By contrast, all the lipoprotein fractions tested, including commercial LDL from Sigma, increased the levels of terminal erythroid maturation observed at days 17 (Figure 6), suggesting that lipoproteins are the factors responsible for improving the enucleation rates in maturation cultures supplemented with human plasma reported by previous studies (van den Akker et al., 2010; Masiello et al., 2013). Although enucleation was not formally investigated in the present manuscript, the observation that cells generated with LDL and VLDL matured readily into ortho-chromatic erythroblasts by days 17 (Figure 6) suggests that these cells, by exhibiting high enucleation rates, represent a better source than erythroblasts expanded with current culture media to generate cRBCs in differentiation cultures.

**FIGURE 7:**
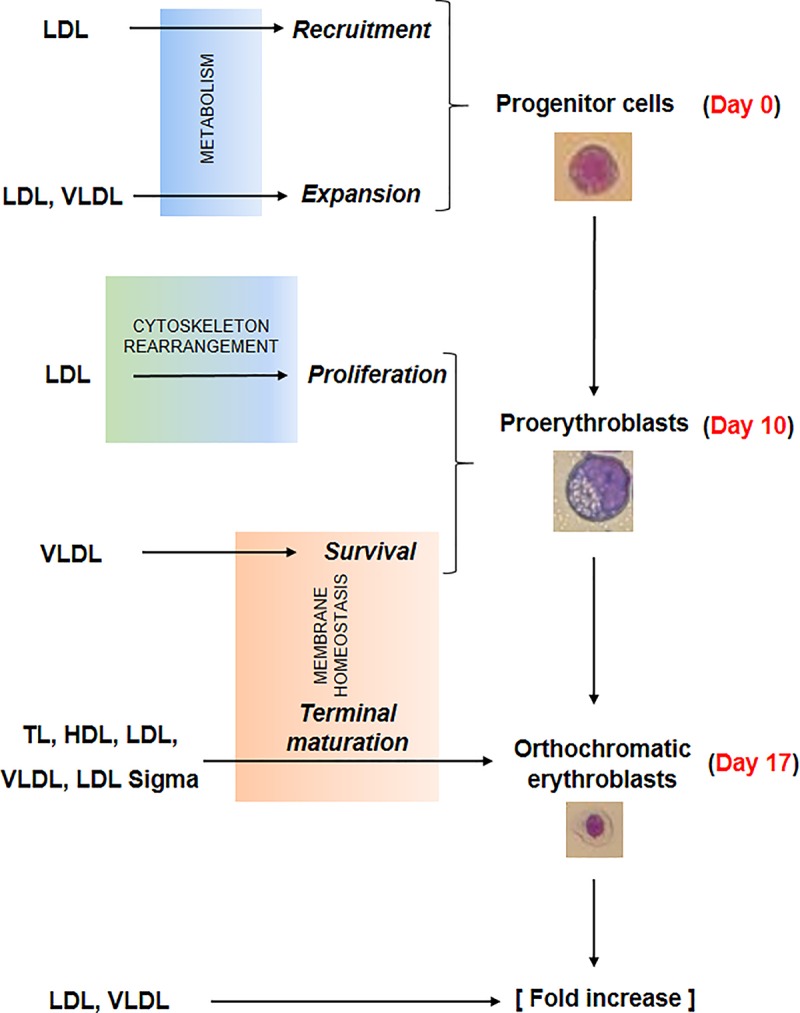
Summary of the effects exerted by the lipoprotein fractions investigated in this study on erythroid differentiation. Recruitment and expansion in liquid culture of the erythroid progenitor cells was improved by LDL and LDL/VLDL, respectively, probably by mechanisms targeting cell metabolism. LDL increased also the proliferation of days 10 proerythroblasts, probably by mechanisms involving rearrangement of the cytoskeleton. Lastly the survival and terminal maturation of the proerythroblasts was favored by LDL and by all the lipoprotein fractions, respectively through mechanisms involving improvement of membrane homeostasis. All these biological effects contributed to the greater erythroid expansion observed in HEMA^def^ supplemented with lipoproteins, expecially when the lipoproteins were represented by LDL and VLDL.

Several events concurred in determining the greater erythroid output observed in proliferation cultures supplemented with lipoproteins. The data presented in this paper provide some clue on the biochemical mechanisms that may underlie some of these events which are summarized in Figure 7.

By expression profiling, erythroblasts generated with and without Dex show differences in only 55 genes, six of which involved in lipid metabolism (Table 1). More specifically, ABCA1, which encodes a cholesterol efflux pump (Litvinov et al., 2018), and HSD17B7, which is involved in cholesterol biosynthesis (Marijanovic et al., 2003), are activated in erythroblasts generated with Dex, suggesting that Dex may promote cholesterol biosynthesis but overall makes these cells cholesterol deficient because of higher efflux rates. Furthermore, Dex inhibits expression of GPAM that encodes an enzyme involved in the synthesis of phosphatidic acid (Wendel et al., 2009), a precursor of TG, suggesting that Dex impairs lipid metabolism in erythroid cells making their membrane biosynthesis highly dependent on lipid absorbed from exogenous sources. Although this interpretation is in partial contrast to other experimental systems such as adipocytes (Lee et al., 2018), the reason of this difference may relay on the specific biological functions exerted by Dex during stress erythropoiesis where it allows the production of greater number of cRBCs by inhibiting terminal maturation and promoting proliferation. In fact, in addition to promoting self-replication (Migliaccio et al., 2002; Zhang et al., 2013), Dex may retain erythroblasts in proliferation by a novel mechanism involving a direct blocking of terminal erythroid maturation through down-modulation of GPAM and, consequently, lower content of phosphatidic acid, the signaling molecule that activates the cytoskeleton rearrangement and the vesicle trafficking (Ferraz-Nogueira et al., 2014; Nguyen et al., 2016) required for this process (see Figure 7).

Our data suggest that lipoproteins reduce the susceptibility of pro-erythroblasts to death induced by growth factor starvation and facilitate their terminal maturation by improving membrane homeostasis. In fact, extensive morphological analyses of erythroblasts generated with Dex indicate that these cells present severe abnormalities of the plasma and mitochondrial membranes suggesting that the lipid bioavailability under current culture conditions is insufficient. The plasma membrane abnormalities came in the forms of pebbles associated with exosome and micro-vesicle formation. Release of micro-vesicles by RBCs involves disturbance of the membrane/cytoskeleton interaction (Leal et al., 2018) and is triggered by Ca^+2^ and protein kinase C activation (Nguyen et al., 2016). The effects of micro-vesicle release on the life span of RBCs *in vivo* are controversial: it may increase, by eliminating hemoglobin precipitates, the life span of sickle RBCs but may decrease that of normal RBCs stored for transfusion (D’Alessandro et al., 2015; Leal et al., 2018). We predict that the membrane abnormalities presented by cRBCs may contribute to reduce their life span *in vitro* and possibly *in vivo* (Giarratana et al., 2005). By argument, we infer that these abnormalities are the direct consequences of cholesterol insufficiency. In fact, the inhibitory function of scramblase activity exerted by cholesterol promotes the phospholipid asymmetry of the membrane that preserves the shape and promotes survival of the erythrocytes (Arashiki et al., 2016). Therefore, by reducing the cholesterol content, Dex may increase the scramblase activity reducing phospholipid asymmetry (and integrity) of the membranes. Genetic evidences for the importance of cholesterol for membrane biosynthesis and RBC survival *in vivo* also exists. Patients with Smith-Lemli-Opiz syndrome which is associated with loss of function mutations in the gene encoding cholesterol biosynthesis DHCR7-7-dehydrocholesterol reductase have low levels of cholesterol and are anemic (Jira et al., 2003). In addition, patients with a rare inherited syndrome leading to synthesis of plant sterols instead than cholesterol experience hemolytic anemia (Wang et al., 2014).

In the mitochondria, membrane abnormalities were observed at the level of the crests and are so severe that predict reduced mitochondrial functions with consequent impaired cell metabolism. They were presented by the majority of the mitochondria independently from their inclusion into autophagic vesicles. Therefore, it is unlikely that these abnormalities were caused by the lytic enzymes in the autophagosomes (Betin et al., 2013). Since phosphatidic acid is also a precursor for cardiolipin, the key phospholipid of the internal mitochondrial membranes controlling their functional integrity, bioenergetics and apoptosis (Paradies et al., 2014; Ikon and Ryan, 2017; Mårtensson et al., 2017), we propose that the membrane abnormalities of the mitochondria observed in erythroblasts generated with Dex are due to reduced cardiolipid content as a consequence of GPAM down-regulation.

Red blood cells express a variety of apolipoprotein receptors, in primis the receptor for ApoA1 and B as demonstrated by the hypocholesterolemia and reduced levels of circulating ApoA1 and B expressed by patients with the acquired erythrocytosis Polycythemia Vera (Fujita et al., 2012). Given the great number of RBCs present in the circulation, these receptors are efficient lipid scavengers contributing to maintain the lipid content of the plasma within physiologic levels preventing atherosclerosis and kidney malfunctions (Wahl et al., 2016; Saraf et al., 2017; Vuorio et al., 2018). The effects on erythroblast expansion sustained by VLDL and LDL described in this study suggests that, in addition to exerting scavenger functions, the lipoprotein receptors expressed by erythroid cells tune lipid availability to prevent possible membrane abnormalities due to impairment of lipid metabolism induced by Dex in RBCs produced under conditions of erythroid stress.

The observation that all the lipoprotein fractions promote terminal maturation while only LDL and VLDL promote erythroblast expansion suggests that the effects exerted by lipoproteins on hematopoietic stem/progenitor cells are mediated by mechanism(s) at least partially different from that exerted during terminal maturation (regulators of lipid bioavailability). These mechanism(s) may be represented by improvement of the overall deficient metabolism caused by Dex (Figure 7) and/or by still to be identified signaling functions exerted by specific protein component of LDL and VLDL. Although further studies are necessary to clarify possible signaling functions of the individual lipoproteins, recent evidences support this hypothesis by indicating that lipoproteins control directly the proliferation of hematopoietic stem/progenitor cells. This proliferation is inhibited by cholesterol bound-HDL through activation of ABCA1 (Yvan-Charvet et al., 2010), a gene found by us activated by Dex (Table 1), while it is promoted by LDL through a mechanism still to be identified (Yvan-Charvet et al., 2010; Feng et al., 2012; Seijkens et al., 2014). These observations may explain why erythroblast expansion is not affected by the presence of HDL and TL, which contain both HDL and LDL, but it is promoted by LDL. Further studies are necessary to clarify the mediators of the effects of LDL on the proliferation of erythroid progenitors and how these mediators interact with the control exerted by HDL/ABCA1 pathway regulated by Dex.

## Conclusion

Overall, the results discussed in this paper highlight the importance of further studies on lipid metabolism and mitochondrial function in erythroblasts and how they are regulated by Dex to increase our understanding on how to improve the functions of RBCs generated *in vivo* under condition of stress. They also demonstrate a novel beneficial effect of LDL and VLDL on recruitment and expansion of erythroid progenitor and precursor cells in the proliferation phase of HEMA^def^, indicating that media formulated with supplements which include clinical grade VLDL and/or LDL will allow the generation of greater numbers of cRBCs that will possible survive longer *in vivo* for transfusion.

## Author Contributions

MZ, CB, LS, and FC performed the experiments, analyzed the data, and wrote the manuscript. GG provided buffy coats from regular blood donations and wrote the manuscript. MLG revised the data and wrote the manuscript. AM designed the experiments, reviewed the data, and wrote the manuscript. All the authors have read the final version of the manuscript and concur with its content.

## Conflict of Interest Statement

The authors declare that the research was conducted in the absence of any commercial or financial relationships that could be construed as a potential conflict of interest.

## Supplementary Material

FIGURE S1 Characterization of the lipoprotein fractions purified from human plasma investigated in the study. **(A)** Diagram of the purification scheme indicating the series of ultracentrifugations used for lipoprotein fractionation. ρ indicates the density of the original plasma or of infranates prepared for ultracentrifugation and is expressed as g/mL. **(B)** Commassie blue and silver staining of the proteins contained in the various lipoprotein fractions separated by gel electrophoresis. The numbers on the top indicate the amount of protein loaded. The position of the molecular weight markers is indicated in kDa on the left while the identity of the bands (predicted on the basis of their size) is indicated by arrows with the corresponding labels (Chapman, 1986). **(C)** Densitometric profile of the bands present in the lanes of the gel stained with Silver presented in **(B)**.

FIGURE S2 Design of the experiments used to evaluate the effects of lipid supplementation on erythroblasts expansion under HEMA^def^ and HEMA^ser^ conditions. As summarized in Supplementary Table S1, end-points of these experiments were represented by expansion of erythroid (liquid cultures, Figure 3) and progenitor (semisolid cultures, Figure 4) cells over time and short-term proliferation (Figure 2), ability to progress along the terminal maturation pathway (Figure 5) and resistance to stress-induced autophagic death (Figure 6).

TABLE S1 list of the end-points analyzed in our study and of their corresponding biological implications.

TABLE S2 Protein and lipid content of the lipoprotein fractions purified from human plasma used in the study. The expected protein/lipid and CH/TG ratios in each fraction on the basis of published data.

TABLE S3 Comparison of the composition of the home made and Sigma LDL fractions.
