# Supplementary material for: Dexamethasone Predisposes Human Erythroblasts Toward Impaired Lipid Metabolism and Renders Their ex vivo Expansion Highly Dependent on Plasma Lipoproteins
Source: Front Physiol. 2019 Apr 4;10:281. doi: 10.3389/fphys.2019.00281 (PMC6458278; doi:10.3389/fphys.2019.00281)
Supplement: FIGURE S1 — Characterization of the lipoprotein fractions purified from human plasma investigated in the study. (A) Diagram of the purification scheme indicating the series of ultracentrifugations used for lipoprotein fractionation. ρ indicates the density of the original plasma or of infranates prepared for ultracentrifugation and is expressed as g/mL. (B) Commassie blue and silver staining of the proteins contained in the various lipoprotein fractions separated by gel electrophoresis. The numbers on the top indicate the amount of protein loaded. The position of the molecular weight markers is indicated in kDa on the left while the identity of the bands (predicted on the basis of their size) is indicated by arrows with the corresponding labels (Chapman, 1986). (C) Densitometric profile of the bands present in the lanes of the gel stained with Silver presented in (B). [file Table_1.pdf]

Table S1: list of end-points used in the paper and of the corresponding biological implications.

Biological events	End-point/ Assay
Cell metabolism and membrane homeostasis	Expression profiling
Total expansion of erythroid cells	Fold increase of cells in liquid culture over time
Number of progenitors recruited in the maturation process	Colony formation Semisolid cultures of primary cells
Generation/survival of progenitor cells in liquid culture	Number of progenitor cells in liquid culture Semisolid cultures of cultured cells over time
Proliferation potential of erythroblasts at day 10 of liquid culture	Proliferation rates of day 10 erythroblasts MTT assay
Apoptotic susceptibility of erythroblasts at day 10 of liquid culture	Apoptotic rates Acrydine Orange after growth factor starvation
Progress of the cultured erythroblasts along the maturation pathway	Flow cytometry for CD36/CD235a expression Maturation profile of cultured cells over time
