# Supplementary material for: Dexamethasone Predisposes Human Erythroblasts Toward Impaired Lipid Metabolism and Renders Their ex vivo Expansion Highly Dependent on Plasma Lipoproteins
Source: Front Physiol. 2019 Apr 4;10:281. doi: 10.3389/fphys.2019.00281 (PMC6458278; doi:10.3389/fphys.2019.00281)
Supplement: FIGURE S2 — Design of the experiments used to evaluate the effects of lipid supplementation on erythroblasts expansion under HEMAdef and HEMAser conditions. As summarized in Supplementary Table S1, end-points of these experiments were represented by expansion of erythroid (liquid cultures, Figure 3) and progenitor (semisolid cultures, Figure 4) cells over time and short-term proliferation (Figure 2), ability to progress along the terminal maturation pathway (Figure 5) and resistance to stress-induced autophagic death (Figure 6). [file Table_2.pdf]

Table S2: **Protein and lipid content of the lipoprotein fractions purified from human plasma used in the study.** The expected protein/lipid and CH/TG ratios in each fraction on the basis of published data (30) is reported in brackets for comparison.

Plasma Fractions	Density (g/mL)	Content (μg/mL)			Ratios	
		Proteins	CH*	TG*	Proteins / (CH+TG)	CH/TG
TL	1.21	24.00	4.40	2.87	3.30	1.53
VLDL	1.006-1.019	5.70 [8]	0.69 [20]	1.92 [55]	2.18 [0.11]	0.36 [0.36]
LDL	1.063	17.40 [20]	2.86 [50]	1.01 [6]	4.50 [0.36]	2.83 [8.33]
HDL	1.21	7.10 [50]	2.87 [20]	1.15 [4]	1.77 [2.08]	2.50 [5.00]

* CH= Cholesterol; TG=triglycerides
