# Supplementary material for: Dexamethasone Predisposes Human Erythroblasts Toward Impaired Lipid Metabolism and Renders Their ex vivo Expansion Highly Dependent on Plasma Lipoproteins
Source: Front Physiol. 2019 Apr 4;10:281. doi: 10.3389/fphys.2019.00281 (PMC6458278; doi:10.3389/fphys.2019.00281)
Supplement: TABLE S1 — list of the end-points analyzed in our study and of their corresponding biological implications. [file Table_3.pdf]

Table S3: Comparison of the composition of the home made and Sigma LDL fractions.

	Composition	
**Plasma Fractions**	**Proteins**	**Lipids**
**Home made LDL***	**82%**	**18%**
**LDL Sigma**<br>(cat. no. LP2-2MG)**	**22-20%**	**78-80%**

* The calculation was made by considering as 100%:
2.86 CH+1.01 TG+17.40 Proteins = 21.27 [µg/mL]

** The composition of LDL Sigma is from:
https://www.sigmaaldrich.com/catalog/product/mm/lp2?lang=it&region=IT
