# Supplementary figures and images for: Dexamethasone Predisposes Human Erythroblasts Toward Impaired Lipid Metabolism and Renders Their ex vivo Expansion Highly Dependent on Plasma Lipoproteins
Source: Front Physiol. 2019 Apr 4;10:281. doi: 10.3389/fphys.2019.00281 (PMC6458278; doi:10.3389/fphys.2019.00281)

A)

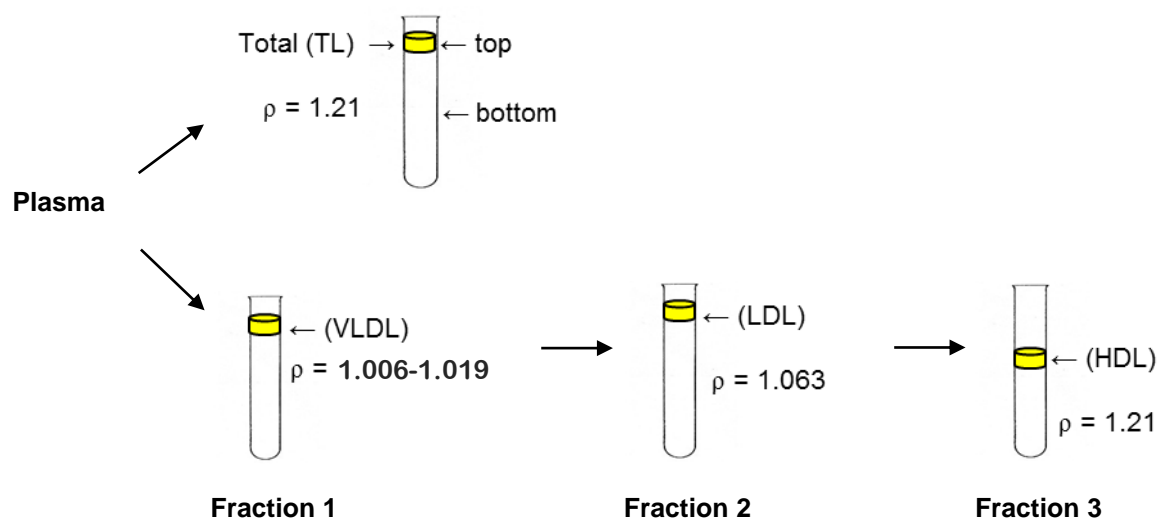

B)

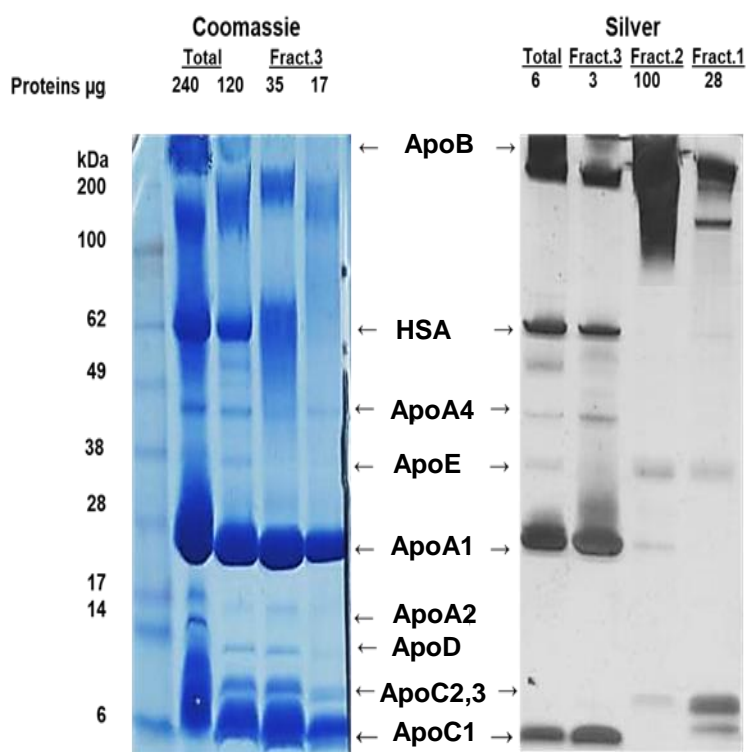

C)

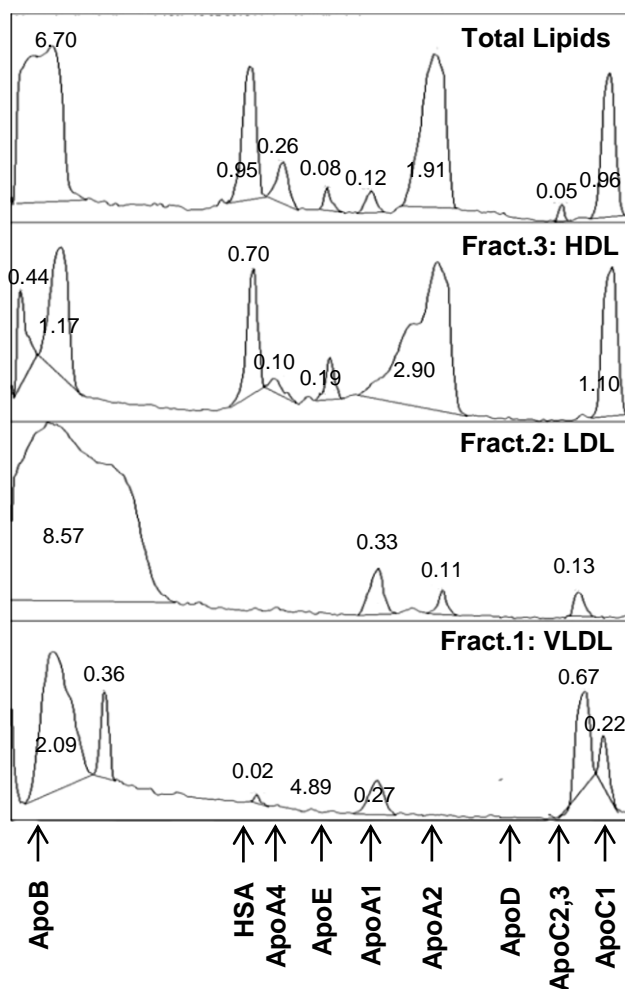

Figure S1

Supplement: TABLE S2 — Protein and lipid content of the lipoprotein fractions purified from human plasma used in the study. The expected protein/lipid and CH/TG ratios in each fraction on the basis of published data. [file Data_Sheet_1.PDF]

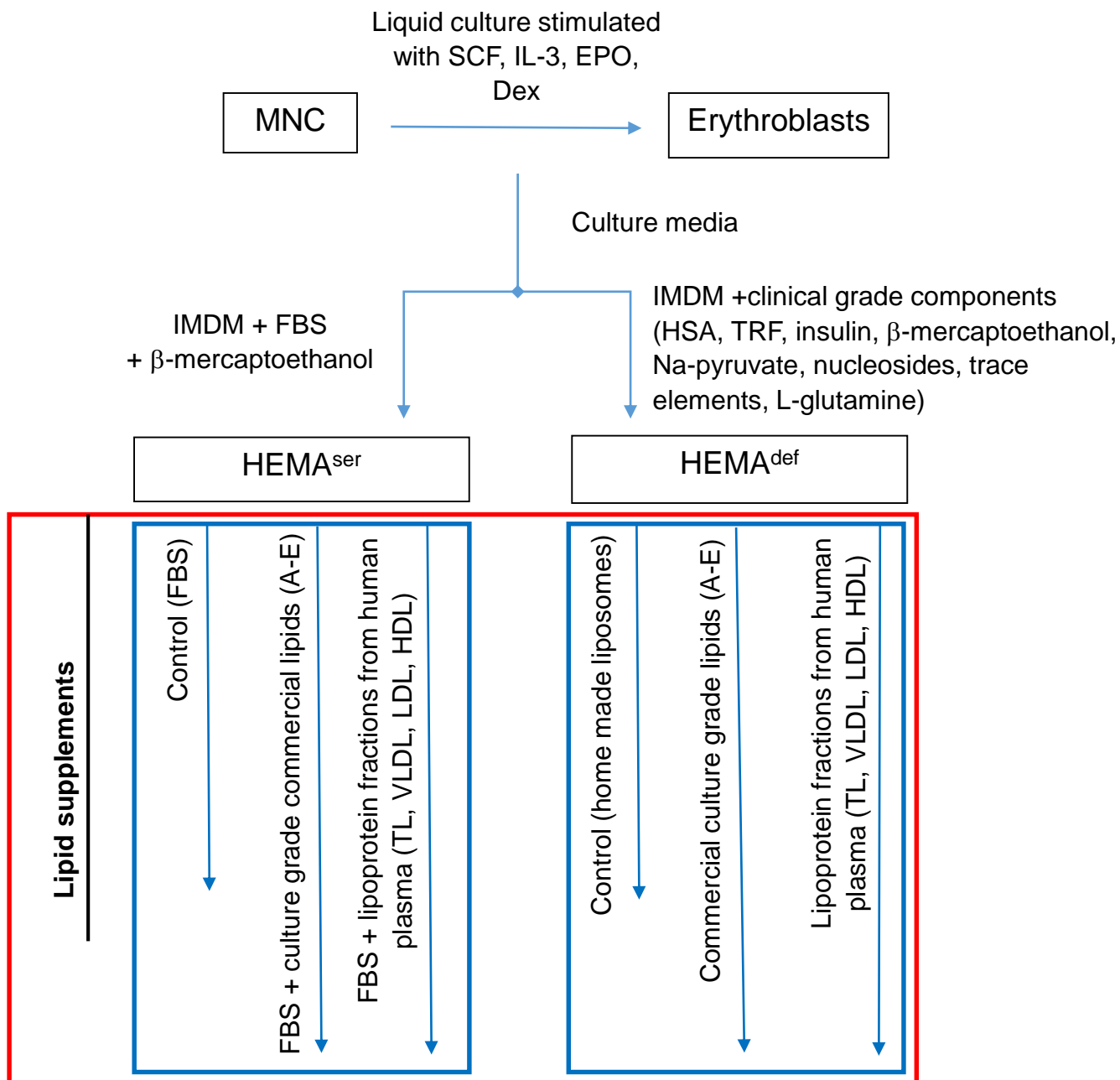

**Figure S2**

Supplement: TABLE S3 — Comparison of the composition of the home made and Sigma LDL fractions. [file Data_Sheet_2.PDF]
